# CoQ_10_ and Cognition a Review and Study Protocol for a 90-Day Randomized Controlled Trial Investigating the Cognitive Effects of Ubiquinol in the Healthy Elderly

**DOI:** 10.3389/fnagi.2019.00103

**Published:** 2019-05-29

**Authors:** Con Stough, Madeleine Nankivell, David A. Camfield, Naomi L. Perry, Andrew Pipingas, Helen Macpherson, Keith Wesnes, Ruchong Ou, David Hare, Judy de Haan, Geoffrey Head, Peter Lansjoen, Alena Langsjoen, Brendan Tan, Matthew P. Pase, Rebecca King, Renee Rowsell, Oliver Zwalf, Yossi Rathner, Matthew Cooke, Franklin Rosenfeldt

**Affiliations:** ^1^Centre for Human Psychopharmacology, Swinburne University of Technology, Melbourne, VIC, Australia; ^2^Faculty of Health, Institute for Physical Activity and Nutrition, Deakin University, Geelong, VIC, Australia; ^3^Wesnes Cognition Limited, Streatley on Thames, United Kingdom; ^4^Department of Psychology, Northumbria University, Newcastle, United Kingdom; ^5^Austin Hospital, University of Melbourne, Melbourne, VIC, Australia; ^6^Baker Heart and Diabetes Institute, Melbourne, VIC, Australia; ^7^East Texas Medical Center and Trinity Mother Francis Hospital, Tyler, TX, United States; ^8^Melbourne Dementia Research Centre, The Florey Institute for Neuroscience and Mental Health, Melbourne, VIC, Australia; ^9^Faculty of Medicine, Dentistry, and Health Sciences, The University of Melbourne, Melbourne, VIC, Australia; ^10^Department of Health and Medical Sciences, Swinburne University of Technology, Melbourne, VIC, Australia

**Keywords:** Coenzyme Q_10_, Ubiquinol, dementia, cognitive decline, cognition, aging, cardiovascular function, RCT

## Abstract

**Introduction**: With an aging population there is an important need for the development of effective treatments for the amelioration of cognitive decline. Multiple mechanisms underlie age-related cognitive decline including cerebrovascular disease, oxidative stress, reduced antioxidant capacity and mitochondrial dysfunction. CoQ_10_ is a novel treatment which has the potential to improve brain function in healthy elderly populations due to established beneficial effects on mitochondrial function, vascular function and oxidative stress.

**Methods and Analysis**: We describe the protocol for a 90-day randomized controlled trial which examines the efficacy of Ubiquinol (200 mg/day) vs. placebo for the amelioration of cognitive decline in a healthy (non-demented) elderly sample, aged 60 years and over. The primary outcome is the effect of Ubiquinol at 90 days compared to baseline on CogTrack composite measures of cognition. Additional cognitive measures, as well as measures of cardiovascular function, oxidative stress, liver function and mood will also be monitored across 30-, 60- and 90- day time points. Data analyses will involve repeated measures analysis of variance (ANOVA).

**Discussion**: This study will be the first of its kind to provide important clinical and mechanistic data regarding the efficacy of Ubiquinol as a treatment for age-related cognitive decline in the healthy elderly with important implications for productivity and quality of life within this age group.

**Clinical Trial Registration**: The trial has been registered with the Australian and New Zealand Clinical Trials Registry (ANZCTRN12618001841268).

## Introduction

The number of older citizens in many western countries is increasing due to advances in medicine and improved living standards (United Nations, 2013). For example, in Australia, the number of citizens aged 65 years or older increased from 14% in 2014 to 16% in 2016. In 1911 this cohort represented 4% of the Australian population and these data are similar in other advanced western countries (ABS, 2019). With increasing age, there are often commensurate impairments to cognitive processes, including attention, memory and processing speed (Wechsler, 1950). This reduction in cognitive function and performance results in a loss of independence and quality of life (Goodwin et al., 1983; Floyd and Hensley, 2002). The rising proportion of the population entering old age has also resulted in a higher rate of age-related neurodegenerative diseases (United Nations, 2013). Consequently, there is an increased burden on government policy and medical spending due to the number of individuals requiring access to aged- and health-care services. Investigating ways to maintain and improve cognition into old age is one way to alleviate some of the burdens on government and health-care services. However, the biological mechanisms behind cognitive aging are complex and yet to be adequately understood.

Cerebrovascular disease is common in the elderly and is a well-recognized contributor to cognitive aging (Tsurio et al., 2003; Vermeer et al., 2003). Two other mechanisms that have been identified as possible causes of cognitive aging are oxidative stress and mitochondrial dysfunction (Golden et al., 2002; Liu et al., 2002; Spindler et al., 2009). Oxidative stress is a key feature of the cognitive aging process, and numerous studies have demonstrated a negative relationship between oxidative stress levels and cognitive performance in humans (Floyd and Hensley, 2002; Markesbery et al., 2005; Simpson et al., 2015). Similarly, mitochondrial dysfunction has been linked to neurodegenerative disease in a range of studies including: animal models, *in vitro* studies of mitochondria levels in patients, post-mortem pathology and neuroimaging (Spindler et al., 2009). It has been proposed that interventions reducing oxidative stress levels and mitochondrial dysfunction can mitigate oxidative damage, slow the rate of aging, and reduce the incidence of neurological disease (Spindler et al., 2009; Simpson et al., 2015).

### Oxidative Stress, Mitochondrial Dysfunction and Aging

A key feature of the cognitive aging process is the body’s increased vulnerability to damage caused by free radicals (Simpson et al., 2015). The increased proliferation of free radicals in old age coincides with a reduction of antioxidant stores within the body. Oxidative stress occurs when there is an imbalance between free radical proliferation, the formation of Reactive Oxygen Species (ROS), and antioxidant stores in the body (Golden et al., 2002; Ryan et al., 2008). If the production of free radicals goes unmitigated, it can cause damage to cellular structures, including neurological structures in the brain (Ryan et al., 2008; Stough et al., 2012). The brain is especially susceptible to oxidative stress due to its high level of oxygen consumption (Simpson et al., 2015). ROS byproducts in the brain are formed during oxygen metabolism, with 95%–98% of all ROS being produced as a result of mitochondrial electron transport chains during aerobic metabolism. Mitochondrial respiration is a key contributor to free radical proliferation, converting 2%–5% of all oxygen into ROS (Golden et al., 2002; Simpson et al., 2015). Hydrogen peroxide (H_2_O_2_), hydroxyl free radical (•OH), superoxide anion (O_2_^−^•) and peroxynitrate (ONO_2_^−^), are molecular species that can cause damage to DNA, protein and membrane lipids in the brain, leading to the death of important neurological structures (Golden et al., 2002; Praticò, 2008; Fiocco et al., 2011; Simpson et al., 2015).

The brain is normally able to defend itself against damage caused by free radicals using antioxidants and systems that remove damaged molecules (Floyd and Hensley, 2002; Golden et al., 2002). Antioxidants act as the first line of defense against oxidative stress and are able to mitigate the damage caused by ROS by donating electrons to free radicals, thereby inhibiting the damage caused. For example, non-enzymatic antioxidants such as ascorbic acid and glutathione (GSH) can reduce the damaging effects of oxidants by converting them to non-radical end products (Simpson et al., 2015). In a healthy body, ROS production is offset by ROS scavenging capacity, however, when free radical proliferation exceeds the body’s ability to scavenge them, an imbalance occurs, creating oxidative stress.

High levels of pro-oxidants such as polyunsaturated fatty acids, transition metals and a high metabolic rate make the brain particularly sensitive to oxidative stress (Praticò, 2008; Simpson et al., 2015). In the brain, pro-oxidants are relatively abundant compared to the levels of antioxidants, such as glutathione peroxidase (GPx) and catalase (CAT), further exacerbating the brain’s vulnerability to oxidative damage (Praticò, 2008; Simpson et al., 2015). In the aging body, protective mechanisms used to combat oxidative stress are even further compromised because of increased free radical proliferation and decreased antioxidant levels (Junqueira et al., 2004; Stough et al., 2012).

The relationship between oxidative stress and normal age-related cognitive decline in humans has been established in numerous studies (Goodwin et al., 1983; Floyd and Hensley, 2002; Morris et al., 2002; Ryan et al., 2008). Additionally, research into oxidative stress in humans report that biological compounds such as oxidized proteins, DNA and lipids are all increased in aging populations (Praticò, 2008; Simpson et al., 2015). Some studies are correlative in nature and have identified an association between oxidative damage to biological compounds and cognitive performance (Uryu et al., 2002, 2007), while others have found positive associations between antioxidant levels and measures of working memory (Masaki et al., 2000; Morris et al., 2002). Alternatively, others have manipulated dietary antioxidant intake and monitored differences in cognitive performance (Goodwin et al., 1983; Perrig et al., 1997). These provide evidence for memory being the cognitive function most affected by oxidative stress, which is congruent with patterns of age-related cognitive decline (Ryan et al., 2008). It is therefore important to expand upon the existing literature of oxidative stress in a non-clinical population to form a better understanding of the biological processes underpinning normal cognitive aging.

Oxidative stress may also play a role in the pathogenesis of Alzheimer’s disease (AD), other neurodegenerative diseases and normal age-associated cognitive decline. Elevated levels of oxidative stress appear concurrently with impairments to cognitive function in patients with AD (Floyd and Hensley, 2002; Montine et al., 2005). Therefore, understanding the biological process by which oxidative stress can impair cognitive performance is crucial to finding ways to fight the insidious effects of cognitive decline and dementia (Praticò, 2008). *In vivo* studies of neurodegenerative diseases such as AD have observed that in the brain, oxidative stress markers such as DNA, protein and lipid peroxidation, are increased in the brain of AD subjects when compared to controls (Floyd and Hensley, 2002; Golden et al., 2002; Praticò, 2008). While human epidemiological studies are mostly consistent with the hypothesis that there is an inverse association between antioxidant levels and intake, cognitive function and AD, randomized clinical trials have not presented significant findings (Praticò, 2008). Sano et al. (1997) conducted a double-blind, placebo-controlled trial administering high-dose vitamin E to AD patients. Vitamin E was found to delay loss of ability to perform basic activities and severe dementia but did not influence the rate of decline of cognitive functions (Sano et al., 1997). In a similar study by Petersen et al. (2005), subjects with a clinical diagnosis of mild cognitive impairment (MCI) receiving antioxidant supplementation showed no difference when compared to controls in the rate of progression from MCI to AD. However, a follow-up longitudinal study by the same authors using magnetic resonance imaging (MRI) showed that changes of the volumes for the hippocampus and entorhinal cortex were less evident in the vitamin E treatment group than in the placebo group (Jack et al., 2008). Thus, the evidence for the oxidative stress theory of aging and neurodegenerative disease is conflicting and there is certainly a need for further research into the area. For example, human studies have used a variety of different antioxidants with varying preparations (natural vs. synthetic), a wide range of dosages, variable length of time and varying measures of cognitive performance. This makes comparison across studies problematic, and for this reason, further research is warranted.

### Coenzyme Q_10_

Coenzyme Q_10_ (CoQ_10_; 2,3 dimethyoxy-5-methyl-6-decaprenyl benzoquinone), or ubiquinone, is a lipid-soluble quinone compound containing a redox active quinone ring and hydrophobic tail (Spindler et al., 2009). The CoQ_10_ found in humans has a polyisoprene chain comprised of 10 isoprene units (five carbons each) with a total of 50 carbons (Crane, 2001). CoQ_10_ is synthesized endogenously and is found in all organs of the human body, but is most densely concentrated in organs that have the highest energy requirements such as the brain, heart, kidney and liver tissue (Spindler et al., 2009). CoQ_10_ is a component in the electron transport chain during mitochondrial oxidative respiration, which generates adenosine triphosphate (ATP). CoQ_10_ accepts electrons from complexes I (NADH-ubiquinone oxidoreductase) and II (succinate dehydrogenase) and is a coenzyme for complex III. Additionally, CoQ_10_ acts as an antioxidant, preventing damage caused by free radicals, including oxidation of lipids within the mitochondrial membrane (Spindler et al., 2009). It should be noted here the different forms of CoQ_10_ in which Ubiquinol is the reduced form of CoQ_10_ and Ubiquinone is the oxidized version of CoQ_10_.

CoQ_10_ also reduces the proliferation of free radicals by activating and increasing anti-apoptotic processes through the expression of mitochondrial uncoupling proteins (UCPs; Spindler et al., 2009). Recent studies have provided evidence for the potential pleiotropic and anti-inflammatory role of CoQ_10_ (see Spindler et al., 2009) by demonstrating its ability to inhibit 1-methyl-4-phenyl-1,2,3,6-tetrahydropyridine (MPTP)—induced interleukin-6 (IL-6), tumor necrosis factor-α (TNF-α), and nuclear factor-κB (NF-κB) expression, thus achieving anti-inflammatory effects (Sharma et al., 2006). Gene expression analysis and cell culture experiments to reduce the activity of inflammatory markers provide further evidence of CoQ_10_’s anti-inflammatory effect *via* gene expression modification (Spindler et al., 2009).

### CoQ_10_ as a Potential Treatment for Cognitive Decline

Mitochondrial dysfunction and oxidative stress have been shown to be major contributors to cognitive decline and the pathogenesis of a number of neurodegenerative diseases (Kidd, 2005). Mitochondrial metabolism is a primary source of ROS (Golden et al., 2002; Simpson et al., 2015). It has been proposed that improved mitochondrial functioning leads to reductions in oxidative damage and thus, reductions in age-related cognitive dysfunction (Liu et al., 2002). Studies investigating the effect of feeding mitochondrial metabolites to rats, have demonstrated the potential to reverse age-associated mitochondrial dysfunction, (Liu et al., 2002). For example, Liu et al. (2002) found that levels of lipid peroxidation, a product of mitochondrial respiration, was significantly higher in old rats compared to younger rats. When old rats were treated with R- α-lipoic acid (LA) antioxidants, lipid peroxidation in these rats was comparable to those seen in young rats. Old rats also had lower levels of oxidative damage to neural structures, including the hippocampus, when LA was given in conjunction with acetyl-L-carnitine (ALCAR), which is necessary in transporting long-chain fatty acids into mitochondria (Liu et al., 2002). The effect of this change was measured by improved performance in the Morris water maze task, demonstrating that a reduction in ROS in older rats is associated with improved memory, spatial learning and hippocampal function (Liu et al., 2002).

Animal studies have revealed that CoQ_10_ supplementation exhibits beneficial anti-oxidative effects, up-regulates mitochondrial function and prevents ATP depletion (Beal, 1999; Ishrat et al., 2006; Spindler et al., 2009). Research by Ishrat et al. (2006) demonstrated that rats that were infused bilaterally with an intra-cerebroventricular injection of streptozotocin (ICV-STZ) experienced significant changes in oxidative damage to their hippocampus and cerebral cortex. Biochemical alternations in the hippocampus and cerebral cortex have been shown to lead to cognitive dysfunction and neurodegenerative diseases such as Alzheimer’s and other dementias (Blokland and Jolles, 1993; Shoham and Youdim, 2000; Hoyer, 2002). Thus, Ishrat et al.’s (2006) study suggests that CoQ_10_ has the potential to play a therapeutic role in the treatment of dementia. Similarly, Beal (1999) demonstrated that CoQ_10_ prevented the loss of dopamine and dopaminergic axons in the striatum of 1-year-old mice treated with MPTP, and Matthews et al. (1998) found that feeding 1-year-old mice 200 mg/kg of CoQ_10_ daily for 1 to 2 months resulted in 30% to 40% increases in mitochondrial concentrations of CoQ_10_ in the cerebral cortex.

In a study on aged transgenic mice overexpressing the Alzheimer presenilin 1-L235P mutation, treatment of 1,200 mg of CoQ_10_ per day for 60 days partially reduced amyloid beta overproduction, as well as intracellular amyloid beta cortical deposits (Yang et al., 2008). Additionally, CoQ_10_ treatment improved markers of oxidative stress such as downregulation of superoxide dismutase (SOD) and increased levels of malondialdehyde in transgenic mice (Yang et al., 2008). Dumont et al. (2011) found that CoQ_10_ treatments in TG19959 transgenic mice models of AD provided protection against plaques and memory loss, as measured by the Morris water maze task. These encouraging observed in animal studies have prompted extensive research into the potential neuro-protective benefits of CoQ_10_ in human clinical populations.

### Cardiovascular Benefits of CoQ_10_

A large body of literature has demonstrated the importance of CoQ_10_ for cardiovascular health (Littarru and Tiano, 2010). CoQ_10_ is highly concentrated in heart muscle due to the energy requirements of these cells. Blood and tissue levels of CoQ_10_ are depleted in congestive heart failure (Boreková et al., 2008) and across a range of cardiovascular conditions. Numerous beneficial effects of CoQ_10_ administration have been observed, including an improvement in symptoms and a reduction in mortality in heart failure (Mortensen et al., 2014), decreases to myocardial thickness (Langsjoen et al., 1993; Adarsh et al., 2008), reduction in hypertension (Rosenfeldt et al., 2007) improvement in cardiac contractility and amelioration of endothelial dysfunction (Tiano et al., 2007) and improved exercise performance (Cooke et al., 2008; Deichmann et al., 2012). In patients with ischemeic left ventricular systolic dysfunction, 8 weeks supplementation with 300 mg daily CoQ_10_ was found to improve mitochondrial function and flow-mediated dilatation; and the improvement of flow-mediated dilatation correlated with the change in mitochondrial function, suggesting that CoQ_10_ improved endothelial function *via* reversal of mitochondrial dysfunction (Dai et al., 2011). In a study of isolated human cardiac mitochondria obtained at the time of cardiac surgery, pre-operative supplementation with CoQ_10_ improved the efficiency of mitochondrial respiration and ATP production (Rosenfeldt et al., 2005). Benefits of 200 mg daily CoQ_10_ to flow-mediated dilatation, a measure of endothelial function, have also been observed after 12 weeks supplementation in statin-treated type 2 diabetic patients (Hamilton et al., 2009). In addition, CoQ_10_ supplementation improved exercise capacity in those treated with statins (Deichmann et al., 2012) and prolonged time to exhaustion in healthy individuals (Cooke et al., 2008). The cardiovascular effects of CoQ_10_ may be attributed to its bio-energetic role and capability to reduce low-density lipoprotein (LDL) oxidation, as well as endothelial dysfunction (Littarru and Tiano, 2010).

### Effects of CoQ_10_ on Brain Function and Cognition

Mitochondrial dysfunction represents a common pathological mechanism of neurodegenerative disorders such as Parkinson’s disease (PD), Huntington’s disease, amyotrophic lateral sclerosis and AD. Due to the role of CoQ_10_ in the mitochondria, there is therapeutic potential for CoQ_10_ administration in these degenerative disorders (Kidd, 2005; Chaturvedi and Beal, 2008). Stamelou et al. (2008) administered CoQ_10_ or placebo to 21 clinically probable progressive supra-nuclear palsy patients for 6 weeks. These researchers identified significant cognitive improvement on a frontal lobe assessment battery, compared to a placebo group, and suggested that CoQ_10_ may have led to a restoration of previously lost functions in individual neurons thereby leading to mild clinical improvement.

In a randomized controlled trial by Li et al. (2015), CoQ_10_ (100 mg tid) and creatine were administered to 75 PD patients with mild cognitive decline (MCI) over an 18-month period. At the end of the treatment period, individuals receiving CoQ_10_ and creatine scored significantly higher on the Montreal Cognitive Assessment (MoCA) together with higher plasma phospholipid levels when compared to individuals in the placebo group. The authors concluded that CoQ_10_ in combination with creatine may delay the rate of cognitive decline associated with PD. In an earlier open-label study by Shults et al. (2002), 300, 600 and 1,200 mg/day CoQ_10_ together with 2,000 IU/day vitamin E was administered to 20 early-PD patients over a 16-month period. A significant dose-dependent reduction in the Unified Parkinson’s Disease Rating Scale (UPDRS) was observed in the patients administered CoQ_10_. However, it is important to note that the UPDRS primarily measures functional impairment rather than cognition.

In relation to AD, RCTs with CoQ_10_ are yet to be conducted, although a few earlier studies have been done involving Idebenone, a synthetic, shorter-chain CoQ_10_ derivative. A double-blind, placebo-controlled multi-center study of 300 AD patients receiving Idebenone revealed a significant improvement in ADAS score after 6 months, as part of a 2-year study (Weyer et al., 1997). Two subsequent trials reported similar improvements with Idebenone treatment slowing the progression of cognitive deficits in AD patients (Gutzmann and Hadler, 1998; Gutzmann et al., 2002).

Only one study has investigated the neurocognitive effects of CoQ_10_ in individuals free from neurodegenerative disease. In this trial, the effects of an acute dosage of 100 mg of CoQ_10_ on quantified EEG (QEEG) was examined in 40–55-year-old hypertensive subjects (Marincola, 1997). In this study, the profile of resting QEEG following CoQ_10_ administration was similar to that of a cognitive enhancer. It is also interesting to note that in a recent case study by Okeahialam (2015) a Nigerian woman with dyslipidemia who had been experiencing statin-induced memory dysfunction, reported resolution of her memory complaints following 4 months of daily CoQ_10_ (100 mg/day) supplementation.

There are several mechanisms *via* which CoQ_10_ may influence cognition. Experimental studies in animal models suggest that CoQ_10_ may protect against neuronal damage produced by ischemia, atherosclerosis and toxic injury (Young et al., 2007). Oral administration of CoQ_10_ increases brain mitochondrial concentrations in animal studies (Bhagavan and Chopra, 2006) and reduces markers of oxidative damage in the cerebral cortex and hippocampus of rats with induced oxidative injuries, whilst also improving cognition (Ishrat et al., 2006). These findings indicate CoQ_10_ can exert neuroprotective effects. Cognitive effects of CoQ_10_ may also be mediated by the cardiovascular system. The association between cardiovascular health and cognitive function has been well established (Breteler et al., 1994). Substances with the capability to modify endothelial function, such as CoQ_10_, may, in turn, be associated with increased cerebral blood flow, enhancing the delivery of oxygen and glucose to the brain (Ghosh and Scheepens, 2009).

In conclusion, there is a good evidence implicating oxidative stress, mitochondrial and microvascular dysfunction in the pathogenesis of age-related cognitive impairment. These pathophysiological processes are all potentially able to be counteracted by CoQ_10_ therapy thus providing the impetus for the current study.

## A Randomized-Controlled Trial of Ubiquinol in the Healthy Elderly

### Aims and Hypotheses

The primary aim of the current study is to examine the chronic effect of 90-day supplementation with CoQ_10_ (Ubiquinol) on cognitive function in a healthy (non-demented) elderly population. The rationale for focussing on older participants is that this population is more sensitive to cognitive improvement given that increasing age is typically associated with cognitive decline-particularly memory and there is currently a scarcity of previous research using sensitive domain-specific measures investigating the cognitive effects of CoQ_10_ in elderly participants without dementia. Further, levels of CoQ_10_ decrease with age, there is increased deterioration in the cardiovascular system with age and finally, there is increased oxidative stress with age and less anti-oxidant protection. All of these indicators suggest that supplementation with CoQ_10_ could improve cognitive function in the healthy elderly. It is hypothesized that 90-day supplementation with Ubiquinol will result in improved cognitive function in healthy elderly participants aged 60 years or older, using a battery of well-validated highly sensitive measures. The potentially positive effects of CoQ_10_ on cardiovascular measures, oxidative stress biomarkers, liver function and mood will also be examined in secondary exploratory analyses.

### Design and Treatment

The study is a randomized, double-blind, placebo-controlled, parallel groups clinical trial with participants randomized to receive Ubiquinol (200 mg/day) or placebo over a 90-day period (Ubiquinol is the reduced form of CoQ_10_). All eligible participants will be assigned to treatment group A or B using a computer-generated random number generator by a disinterested third party. Ubiquinol and placebo will be matched in appearance. Randomization codes will be kept in a password protected computer file and only opened in case of emergency.

### Participants

A total of 128 healthy elderly participants (52 per group) aged 60+ years will be randomized. The sample size was selected on the basis of power analysis with G*Power 3.1.9.2, which revealed that with two treatment groups and two primary time points and specifying a significance level of *p* < 0.05, there is an 80% chance of detecting a small-medium effect size (cohen’s *f* = 0.14) for the time × treatment group interaction. Inclusion criteria will include being male or female aged 60 years or older, fluent in written and spoken English, normal or corrected-to-normal eyesight and good general health on the basis of medical history. Exclusion criteria will include being a current smoker, or having a history of the following medical conditions: diabetes, epilepsy, PD, dementia, stroke (or other neurological conditions), traumatic brain injury, current anxiety, depression, alcohol or substance dependence, endocrine, gastrointestinal or bleeding disorder, and uncontrolled hypertension (as determined by a systolic blood pressure >160 mm Hg or diastolic blood pressure >90 mm Hg). Participants will also be excluded if they are currently using over-the-counter herbal or nutritional supplements with known effects on cognition and mood, anti-coagulant drugs, anti-depressants (e.g., selective-serotonin reuptake inhibitors) or anxiolytics (e.g., benzodiazepines).

### Procedure

Testing will be conducted at the Centre for Human Psychopharmacology (CHP), Swinburne University of Technology, Melbourne, VIC, Australia over a 12-month period. Initial telephone screening will be conducted over the phone, and participants determined eligible for inclusion in the study will be booked in for a Screening visit (Day-7). At the screening visit, participants will attend CHP where informed consent will be obtained and their final eligibility will be confirmed by an experienced research assistant. Demographic information will be collected (age, height, weight, education, gender, handedness, English language skills and employment status) and participants will be screened for dementia using the Memory Assessment Clinic-Q (MAC-Q; Folstein et al., 1975) whereby a score ≥19 will be required for inclusion in the study. Participants will then complete a practice session including all CogTrack tasks and other cognitive assessments.

Baseline testing (Day 0) will be completed within 14 days of the screening and practice visit. Upon arrival at the CHP, the participant will provide a fasting blood sample and eat a standardized breakfast. Brachial blood pressure will be recorded with the participant lying down after a 5-min rest period. Measurements will be calculated using an automatic sphygmomanometer and an appropriately sized cuff. Participants will undergo an assessment of cardiovascular function using the Sphygmocor and have a cardiovascular wristwatch fitted (BPro, to measure blood pressure over 24 h). During this time, participants will complete an Automated Self-Administered 24-Hour Dietary Assessment Tool (ASA24) for 3 days food consumed, portion sizes and nutrient composition. Participants will be asked whether they have experienced any adverse events since the last visit. They will be required to avoid eating from 10 pm the night before and avoid the use of alcohol for 24-h prior to the session. Participants will then complete the Profile of Mood States (POMS; McNair et al., 1971), Prospective and Retrospective Memory questionnaire (PRMQ), CogTrack battery and other cognitive tasks (see Table 1). Last, participants will complete a submaximal 6-min walk test (6 MWT). At the end of baseline testing, participants will be supplied with 90 days of their allocated treatment. They will be required to consume two capsules daily for the 90-day study duration, and monitor their compliance using a treatment log. After 30-days and 60-days of treatment, participants will complete online questionnaires and cognitive tasks (ASA24 × 3, POMS, PRMQ and CogTrack) from home. Each participant will be sent a link, *via* email, that will direct them to the online portal where their assessment tasks will be completed. At the final 90-day visit, participants will again attend CHP and follow the same procedure as for Day 0 with completion of ASA24 3 days prior. Participants will be advised to maintain their usual diet and exercise routines. The timing of testing for all primary and secondary outcome measures across study visits is displayed in Table 1.

**Table 1:** Summary of primary and secondary outcomes across study time points.

Study Event	Day −7	Day 0	Day 30	Day 60	Day 90
Screening (*Medical Hx and screening, demographics, Mac-Q*), practice of cognitive tasks		X			
Randomization to CoQ_10_ or placebo treatment group, provided capsules.	X				
CogTrack battery	X	X	X	X	X
Prospective and retrospective memory questionnaire (PRMQ)	X		X	X	X
Profile of Mood States (POMS)	X		X	X	X
Wechsler memory scales (WMS-IV)	X	X			X
Neuropsychological battery (*DSST, WAIS-III Digit Span, TMT-A/B, IT, RAVLT*)	X	X			X
Dietary Assessment tool (ASA24)	X	X			X
Cardiovascular Function (*Brachial blood pressure, SphygmoCor measures*)	X				X
Biochemical assessment (fasting blood sample for F2-isoprostanes, liver enzymes, CoQ_10_, C reactive protein)	X				X
Safety (adverse event reporting)	X		X	X	X
Treatment compliance log	X		X	X	X
6 minute walk		X			X
BPro Wrist watch		X			X

### Primary Outcome

The primary study outcome is the effect of CoQ_10_ (Ubiquinol) supplementation on cognitive performance at 90-days, using the composite measures from the CogTrack battery (Wesnes et al., 2017a,b, 2018; Watson et al., 2018). CogTrack is a well-validated and highly sensitive online computerized battery for assessing cognitive function across domains of attention, concentration and vigilance, working memory and executive control, and episodic/declarative memory. Scores from the individual CogTrack tests are used to calculate five index scores: attentional intensity index, sustained attention index, working memory capacity index, episodic memory capacity index, and speed of retrieval index.

### Secondary Outcomes

The secondary outcomes include effects of CoQ_10_ supplementation on a range of cognitive, mood, cardiovascular function and endurance, and biochemical outcomes: (i) 30- and 60-day CogTrack composite scores; (ii) other cognitive measures at 90-days including the Wechsler memory scales, revised (WMS-IV; Wechsler, 1997), Digit symbol substitution test (DSST) and Digit Span test from the Wechsler Adult Intelligence Scale III (WAIS-III; Wechsler, 1997), Trail Making Tests (A and B), Inspection time (IT), and Rey’s Auditory Verbal Learning Test (RAVLT; Rey, 1964); (iii) 30-, 60- and 90-day Profile of Mood States Questionnaire (POMS; McNair et al., 1971) score; (iv) 30-, 60- and 90- day PRMQ (Crawford et al., 2003) score; (v) 90-day Cardiovascular function (brachial blood pressures and aortic blood pressures and reflection calculated using Sphygmocor); (vi) 90-day 6 MWT, (vii) 90-day Oxidative Stress biomarkers [F2-isoprostanes, Glutathione peroxidase (GPX), High-sensitivity C-Reactive Protein]; and (vii) 90-day Liver Function assessments (total bilirubin, total protein, albumin, globulin, alkaline phosphatase, alanine aminotransferase, gamma glutamyl transferase).

In addition, participants will be required to complete online diet recall assessments on six occasions in their own home. This data will be used to understand whether diet plays a role in any of the outcome measures. The cardiovascular measures provide mechanistic variables in which may assist us in better understanding any cognitive benefits of the treatments and the liver enzymes will assist us in monitoring participant safety.

### Cardiovascular and Exercise Assessments

Participants will be screened according to Exercise and Sports Science Australia’s Adult Pre-Exercise Screening Tool, and will only be permitted to participate if they are deemed to be of low risk. They will have no known cardiovascular disease, and to the best of their knowledge, be able to perform the exercise task. Participants will be familiarized will all testing procedures. Participants will complete the following battery of tests at baseline (Week 0) and Week 12.

### 24-h Radial Blood Pressure

Participants will be fitted with a BPro wristwatch at their baseline visit (Day 0) and final visit (Day 90) and will be required to wear this for 24 h. The radial artery position will be located and BPro fitted accordingly. The device will be calibrated by measuring the participant’s blood pressure using an automatic sphygmomanometer and appropriately sized cuff three times at the time of fitting the BPro, and the average of these three measurements will be entered into the wristwatch. The BPro may need to be secured in place for some participants using plasters and an “anchor” device. This will only be done if comfortable for the participant.

The BPro captures blood pressure over 24 h at the wrist, eliminating the possibility of a “white coat” effect and providing more reliable blood pressure estimates. It is convenient to use, light weight, non-invasive, non-disruptive, cuffless, and takes a BP reading every 15 min over 24 h. The design of the watch provides a constant force of applanation on the radial artery, thereby capturing the waveforms of the radial artery. The device measures systolic and diastolic blood pressure, heart rate, central aortic systolic pressure (CASP) and 24 h blood pressure patterns. Where there are less than 45 recordings available during a 24 h period the data will be excluded from analysis.

### Six Minute Walk Test

The 6 MWT is a submaximal exercise test that entails measurement of distance walked over a span of 6 min. The 6-min walk distance (6 MWD) provides a measure for integrated global response of multiple cardiopulmonary and musculoskeletal systems involved in exercise. The 6 MWT will be performed in accordance with the published guidelines by the American Thoracic Society (ATS). The test has been marked at 3-m intervals so that an accurate measurement of the walking distance can be performed. Chairs are available at 30-m intervals in case the patients become so symptomatic that they have to stop and sit. Participants will rest comfortably for 10 min prior to the test. During this time blood pressure and heart rate should be measured and potential contraindications assessed. Prior to the test commencing, the participant will stand up and rate his/her dyspnea and fatigue. The Borg scale (Rating of Perceived Exertion) will be used for this. The participant will begin the test with the supervisor/researcher using standardized phrases and an even tone for encouragement at the completion of each minute of the test. The participant will be allowed to rest during the test, but the clock will continue. If the participant cannot continue walking, the test will be stopped and the distance covered recorded. At the conclusion of the test, the participant will be asked to rate his/her dyspnea and fatigue levels and the reason for stopping the test.

### Statistical Analysis

The primary analysis will involve an intention-to-treat (ITT) examination of the effects of treatment (CoQ_10_ Ubiquinol vs. placebo) on CogTrack index scores (attentional intensity index, sustained attention index, working memory capacity index, episodic memory capacity index, and speed of retrieval index) at 90-days compared to baseline. Repeated measures analysis of variance (ANOVA) will be used to examine treatment and study visit × treatment interaction effects, using a subject-specific random intercept term, treatment group as a between-group factor (CoQ_10_, placebo) and study visit as a repeated within-group factor (baseline, 90-days). Pearson’s *r* correlations will be used to explore the relationships between demographic variables (i.e., age, weight, height, education, gender) and CogTrack composite scores, and in the case of significant associations (*p* < 0.05), demographic variables will be included as covariates in the analysis.

Similar statistical techniques will be used to investigate the effects of treatment on secondary outcomes, as well as the CogTrack composite scores at 30- and 60-day time points. Results will be presented as effect sizes using 95% confidence intervals. Overall compliance to treatment will be analyzed by counting each participants remaining supplements at the completion of the trial.

## Discussion

With an aging population, there is an important need for the development of effective treatments for the amelioration of cognitive decline. CoQ_10_ is a novel treatment which has the potential to improve brain function in healthy elderly populations due to established beneficial effects on mitochondrial function and oxidative stress. However, there is currently a scarcity of previous human clinical trial research to explore the cognitive effects of CoQ_10_ in the healthy elderly. The current study will be the first of its kind to provide important clinical data regarding the efficacy of CoQ_10_ as a targeted treatment for age-related cognitive decline, and aid in increasing productivity and quality of life in this age group. There are a number of issues that the current study, like all clinical trials, will not be able to address. For instance, the duration of treatment? Although based on previous research and our power analysis we expect to see significant changes after 3 months of supplementation, it is possible that this may not be long enough to see the effect of treatment on cognitive function. Like many nutraceuticals, the effect may be cumulative over time so that longer durations of administration lead to greater functional effects. Another issue is the age of the participants. We will test a relatively wide range of ages for an older demographic but it is also possible that a greater treatment effect could be observed where cognitive decline is greatest i.e., in an even older cohort. Finally, a more sensitive cohort in terms of cognitive function may be a sample with MCI or specific memory deficits. In addition to the main outcome variables, we will also provide information on adverse effects, both serious adverse effects and adverse effects due to the treatment. This information will be helpful to health care practitioners and the general community in terms of the potential cost-benefit ratio of the treatment. Finally, we intend to publish the results of the trial in peer-review relevant journals. There are no restrictions in our ability to disseminate positive or negative results.

## Ethics Statement

The protocol was approved by the Swinburne University Human Research Ethics Committee. SHR Project 2018/323. All subjects will provide written informed consent in accordance with the Declaration of Helsinki.

## Author Contributions

CS, RO, FR contributed to the grant proposal. CS, MN, DC, NP, AP, HM, KW, RO, DH, JDH, GH, PL, AL, MP, RK, RR, YR, MC and FR contributed to the design and methodology. CS, DC, BT, OZ and FR contributed to initial drafts of the manuscript.

## Conflict of Interest Statement

KW is CEO of CogTrack. The remaining authors declare that the research was conducted in the absence of any commercial or financial relationships that could be construed as a potential conflict of interest.
